# Regional myocardial motion in patients with mild cognitive impairment: a pilot study

**DOI:** 10.1186/s12872-018-0824-2

**Published:** 2018-05-02

**Authors:** Heng Ma, Jun Yang, Haizhu Xie, Jing Liu, Fang Wang, Xiao Xu, Wei Bai, Kai Lin

**Affiliations:** 1 Department of Radiology, Yuhuangding Hospital, Qingdao University School of Medicine, 20 E Yuhuangding Rd, Yantai, 264000 Shandong China; 2 Department of Radiology, Northwestern University Feinberg School of Medicine, 737 N Michigan Ave, Ste 1600, Chicago, IL 60611 USA

**Keywords:** Mild cognitive impairment, Cardiac MRI, Regional myocardial motion

## Abstract

**Background:**

Cardiovascular disease (CVD) is a risk factor for cognitive impairment in the elderly. Manifestations of subclinical CVDs can be found in patients with cognitive impairment. The aim of the present study was to test the hypothesis that patients with mild cognitive impairment (MCI) have different magnetic resonance imaging (MRI)-derived regional myocardial motion indices compared with healthy controls.

**Methods:**

Eleven MCI patients (age, 65.5 years ±5.9; range, 55–81 years old) and 11 sex−/age-matched healthy volunteers were enrolled. All of the participants underwent a head MRI and cardiac MRI. Global cortical atrophy (GCA) was graded on the head MRI. The left ventricular ejection fraction (LVEF) and regional strain, strain rate, displacement and velocity were measured on cine images. The GCA scores, global cardiac function and regional myocardial motion indices were compared between MCI patients and healthy controls using the t-test.

**Results:**

MCI patients had a higher GCA score than healthy controls (*p* = 0.048). However, there was no significant difference in LVEF between MCI patients and controls. Compared to healthy controls, MCI patients had a lower peak radial strain (29.1% ± 24.1% vs. 46.4% ± 43.4%, *p* < 0.001), lower peak diastolic radial strain rate (3.2 ± 2.4 s^− 1^ vs. 6.0 ± 3.0 s^− 1^, *p* < 0.001), lower peak diastolic circumferential strain rate (2.5 ± 2.1 s^− 1^ vs. 3.2 ± 2.1 s^− 1^, *p* = 0.002), lower peak systolic radial displacement (4.2 ± 2.2 mm vs. 5.2 ± 3.3 mm, *p* = 0.002), lower peak diastolic radial velocity (31 ± 18 mm/s vs. 45 ± 33 mm/s, *p* < 0.001), and lower peak diastolic circumferential velocity (178 ± 124 degree/s vs. 217 ± 131 degree/s, *p* = 0.005).

**Conclusion:**

MRI-derived regional myocardial strain, strain rate and velocity were found to be different between MCI patients and healthy controls. Regional myocardial motion indices have the potential to become novel quantitative imaging biomarkers for representing the risk of neurodegenerative disorders, such as Alzheimer’s disease (AD).

**Electronic supplementary material:**

The online version of this article (10.1186/s12872-018-0824-2) contains supplementary material, which is available to authorized users.

## Background

Alzheimer’s disease (AD) is the most common cause of cognitive impairments, including mild cognitive impairment (MCI) and dementia, in older adults [1]. Cardiovascular disease (CVD) has been considered a prominent contributor to AD in the elderly [2, 3]. An intuitive mechanistic link between CVD and AD is chronic brain hypoxia due to progressive heart dysfunction [4]. Nonetheless, the causal relationship between CVD and AD is still not fully understood and there is no consensus on whether treating CVDs can reduce AD risk. “Health United States Report 2016” (www.cdc.gov/nchs/data/hus/hus16.pdf) showed that, from 2000 to 2015, deaths per 100,000 people (after age adjustment) from CVDs decreased substantially (from 257.6 to 168.5) while deaths from AD increased dramatically (from 18.1 to 29.4). *These outcomes suggest that advancements in cardiovascular treatment strategies could successfully extend the lives of CVD patients but did not directly improve AD outcomes. By the time CVD is detected and treated, its impact on the brain might have already been irreversible, suggesting that early intervention for subclinical CVD is critical to prevent AD* [1]*.* Unfortunately*, conventional quantitative cardiovascular* indices of global cardiac function, such as left ventricular ejection fraction (LVEF), are insufficient to link subclinical CVD to AD [5, 6]. A logical explanation is *that myocardial ischemia and* dysfunction in the early stage of CVD can occur at a regional level and might not initially impact the global cardiac function. Therefore, cardiac imaging biomarkers that can better bridge subclinical CVD to AD are highly desired.

Recently, quantitative biomechanical indices acquired using magnetic resonance imaging (MRI), such as strain, strain rate, displacement and velocity, have been adopted to comprehensively describe abnormal myocardial motion patterns [7]. Compared with global indices, regional myocardial motion indices seem to be more appropriate candidates for describing subtle cardiac changes affiliated with subclinical CVDs. However, whether regional myocardial motion indices have the potential to indicate the risk of AD-related cognitive impairments has not been fully demonstrated. To address this unmet clinical need, we investigated regional myocardial motion indices in MCI patients who do not have documented CVDs using quantitative cardiac MRI. The aim of the present study was to test the hypothesis that MCI patients have different regional myocardial motion indices compared with healthy controls.

## Methods

### Participants

This prospective study was approved by the local ethics review board and written informed consent was obtained from all of the participants. From 2012 to 2017, the medical records of MCI patients in our institution were reviewed. All of the MCI patients were clinically diagnosed at our institution by experienced neurologists according to the guideline published by National Institute on Aging-Alzheimer’s Association workgroups in 2011 [8]. Specifically, the diagnosis of MCI was based on the following criteria: 1) reasonable concern from the patient or his/her relatives regarding a recent change in cognition/behavior compared with the patient’s previous level; 2) the impairment of cognitive in at least one major domain, such as executive function, episodic memory, attention, visuospatial capability, or language skills. The impairment should be beyond the patient’s educational background and age scale; and 3) the patients should preserve independence in daily abilities with minimal aid or assistance. There is no evidence of significant impairment in social or occupational functioning and he/she should not be demented. The inclusion criteria for participants were: 1) ≥ 55 years old; 2) without documented structural CVDs; 3) without dementia or other psychiatric diseases; 4) without stroke or brain tumor; and 5) without other life threatening conditions that can significantly affect the cardiovascular system, including kidney dysfunction and rheumatologic diseases. The exclusion criteria were: 1) participants with contradictions of MRI. In total, 11 patients with MCI and 11 sex- and age-matched (± 2 years of difference allowed) healthy volunteers were enrolled in this study. The control group consisted of individuals without either history or signs of structural CVDs or cognitive impairments. See Table 1 for demographic information of the participants.

**Table 1:** General Characteristics of MCI Patients and Controls*

	MCI patients (*n* = 11)	Healthy controls (*n* = 11)	*p* values*
Age (years)	65.5 ± 5.9	66.4 ± 8.3	NS
Male (%)	6 (55)	6(55)	NS
Hypertension (%)	2(18)	2(18)	NS
Diabetes mellitus (%)	3 (27)	4(36)	NS
Smokers (%)	2 (18)	3(27)	NS
Systolic blood pressure (mmHg)	134.6 ± 8.9	126.3 ± 15.6	NS
Diastolic blood pressure (mmHg)	80.3 ± 7.6	82.5 ± 9.2	NS
Body mass index (Kg/m2)	26.8	25.7	NS

* “NS” indicates that no statistically significant difference was found between the two groups

### MR protocols

All of the participants were imaged with a 3.0 T clinical MRI scanner (Signa EXCITE, GE Healthcare, Waukesha, WI, USA) by two certified MRI technicians. The cardiac MRI and head MRI were consequently performed on the same day with a 4-element phased-array surface coil and a head coil, respectively. Blood pressure (BP), body weight and height were measured for each participant immediately before MRI scans. Related health information for assessing traditional cardiovascular risks was collected by checking the medical history.

### Cardiac MRI procedure

After localization of the heart, cine images were acquired by using a Fast Imaging Employing Steady-state Acquisition (FIESTA) sequence at two-chamber, three-chamber and short-axis views. The short-axis cine images covered the heart from base to apex with 10–12 slices. The imaging parameter included: TR/TE = 3.6/1.6 ms, field of view (FOV) = 380 × 380 mm, matrix = 512 × 512, slice thickness = 8 mm. Twenty retrospective phases were constructed within a cardiac cycle.

### Head MRI procedure

A three-plane localizer was used to prepare the imaging planes. A fast spin echo (FSE) sequence was run on axial and sagittal views to obtain T1 weighted images (T1WI) and T2 weighted images (T2WI) covering the whole brain. The imaging parameters included: T1WI: TR/TE = 750/25 ms, FOV = 200 × 200 mm^2^, matrix = 512 × 512, slice thickness = 8 mm. T2WI: TR/TE = 3500/100 ms, FOV = 200 × 200 mm^2^, matrix = 512 × 512, slice thickness = 8 mm.

### MR image analysis

#### Head MRI analysis

All of the images underwent regularly checks by reviewer #1 (H.M, with 10-year experience in clinical radiology) before cardiac MRI analysis was started. Global cortical atrophy (GCA, four-point scale, score 0–3) scores were then graded semi-quantitatively by reviewer #1 based on T1WI and T2WI images using criteria from the existing literature [9]. Grades of GCA were determined by judging the widening of the sulci and gyral volume loss by reviewer #1. Normal sulci received a GCA grade of 0, slight widening of sulci was considered to have a GCA of 1, gyral volume loss was assigned a GCA of 2 and prominent sulci widening with substantial volume loss was categorized as a GCA of 3.

#### Cardiac MRI analysis

All of the cardiac images were analyzed using Circle CVI42 software (Calgary, Canada) on a dedicated workstation (Dell, Precision T1650, USA). After short-axial and four-chamber cine images were loaded, reviewer #1 manually traced the endocardial and epicardial borders of the left ventricle (LV) to define region of interest (ROI) at the end-systolic and end-diastolic phases, respectively. MRI-derived global cardiac function and regional myocardial motion indices, such as LVEF, strain, strain rate, displacement and velocity, were automatically calculated based on the ROIs. Regional motion indices in the radial and circumferential directions were mapped on an AHA 16-segment LV model. Long-axis motion indices were mapped on a 6-segment LV model. Measures for each participant were output as a single Microsoft Excel spreadsheet for further analysis.

#### Quality control (QC) for measurements of regional myocardial motion indices

Reviewer #1 re-analyzed the data of all of the cases after a one-month interval to test the intra-observer agreement in measurements of regional myocardial motion indices. Reviewer #2 (K.L, with 13-years of experience in clinical radiology) independently analyzed all of the cases with the same workflow to test inter-observer agreement of GCA grading and myocardial motion measurements.

### Statistics processing

Data are presented as the mean ± one standard deviation (SD). GCA scores, LVEF, and regional myocardial motion indices (16 data points on short-axis views and 6 data points on four-chamber views) were compared between two participant groups using the t-test. Pearson’s correlation coefficient (r) was applied to test correlations between GCA scores and cardiac motions indices. Bland-Altman plots were used to present intra- and inter-observer variations for measuring regional myocardial motion indices. Inter-observer agreement of GCA grading between two reviewers were evaluated using intra-class correlation coefficient (ICC). Statistical analysis was performed using SPSS software (Version 22.0, IBM, USA). A *p* value < 0.05 was considered to demonstrate statistical significance.

## Results

**Fig. 1:**
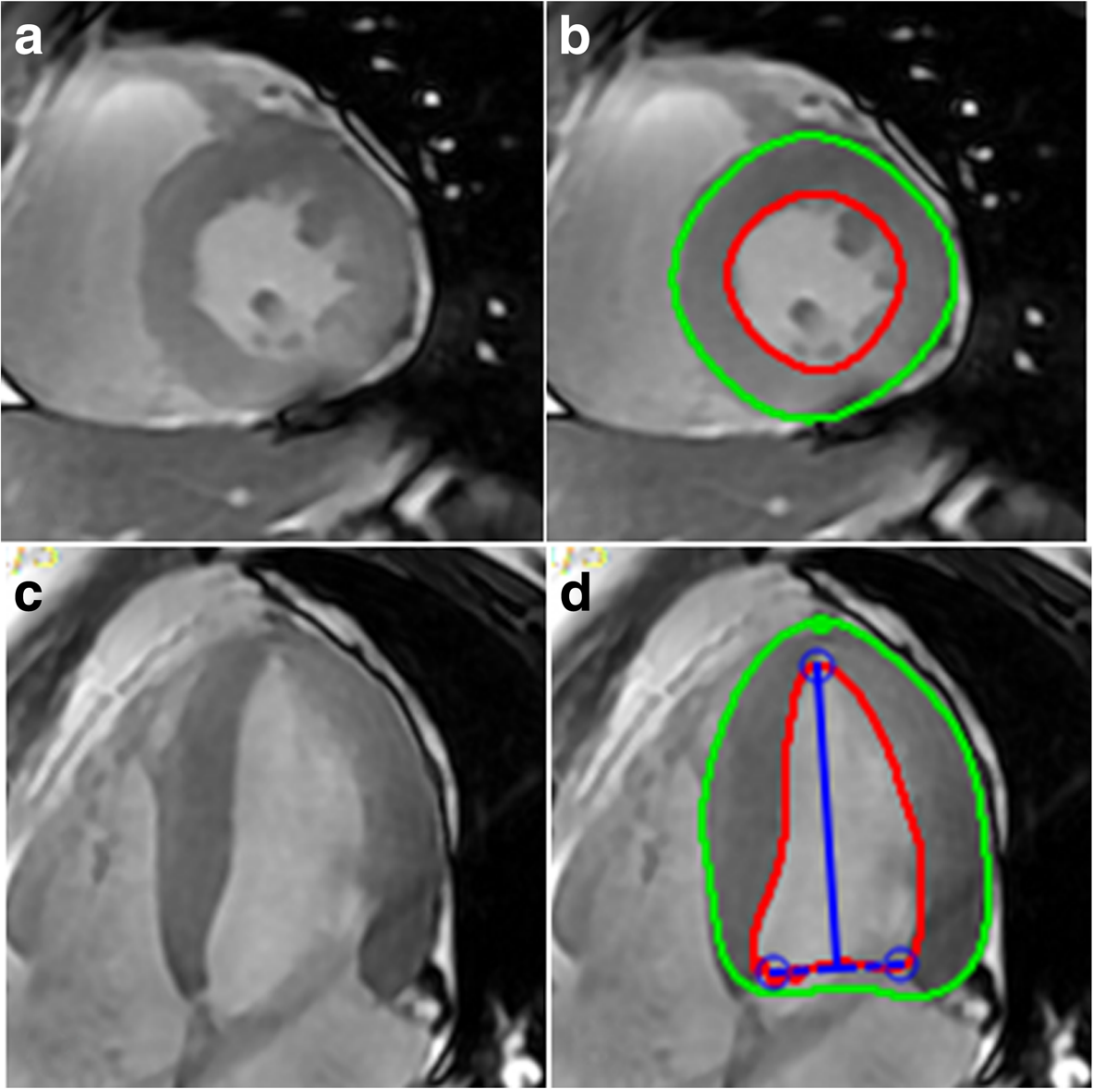
Contours on cine images for measuring regional myocardial motion indices. **a** Short-axis view of LV without contours. **b** Short-axis view of LV with contours. **c** Four-chamber view of LV without contours. **d** Four-chamber view of LV with contours

All 22 participants underwent cardiac and head MRI successfully. The image quality of all the participants was eligible for quantitative study. No significant differences in cardiovascular risks were found between the two participant groups (Table 1). Head MRI showed that MCI patients seemed to have a higher average GCA score than healthy controls (1.18 ± 0.4 vs. 0.82 ± 0.4, *p* = 0.048). However, there were no significant differences in LVEF between MCI patients and controls (52% ± 11% vs. 55% ± 10%, *p* = 0.277). Figure 1 shows the contours on the myocardial borders of the LV.

**Fig. 2:**
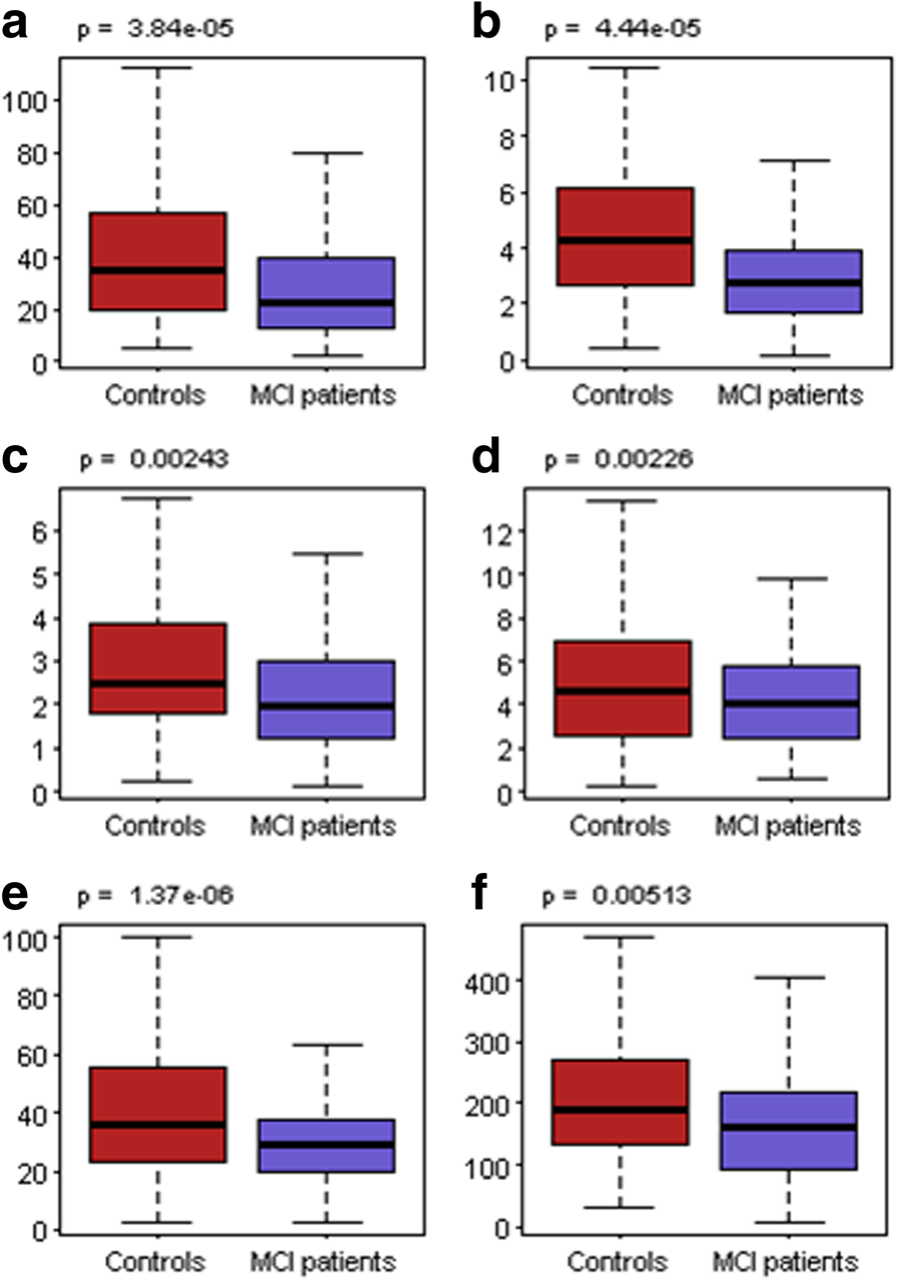
Differences in regional myocardial motion indices between MCI patients and healthy controls (*p* < 0.05). **a** Peak radial strain (%). **b** Peak diastolic radial strain rate (s^− 1^). **c** Peak diastolic circumferential strain rate (s^− 1^). **d** Peak radial displacement (mm). **e** Peak diastolic radial velocity (mm/s). **f** Peak diastolic circumferential velocity(degree/s)

Compared to healthy controls, MCI patients had a lower peak radial strain (29.1% ± 24.1% vs. 46.4% ± 43.4%, *p* < 0.001), lower peak diastolic radial strain rate (3.2 ± 2.4 s^− 1^ vs. 6.0 ± 3.0 s^− 1^, *p* < 0.001), lower peak diastolic circumferential strain rate (2.5 ± 2.1 s^− 1^ vs. 3.2 ± 2.1 s^− 1^, *p* = 0.002), lower peak systolic radial displacement (4.2 ± 2.2 mm vs. 5.2 ± 3.3 mm, *p* = 0.002), lower peak diastolic radial velocity (31 ± 18 mm/s vs. 45 ± 33 mm/s, *p* < 0.001), and lower peak diastolic circumferential velocity (178 ± 124 degree/s vs. 217 ± 131 degree/s, *p* = 0.005). See Fig. 2. However, no significant differences in regional long-axis myocardial motion indices between the two participant groups were found.

**Fig. 3:**
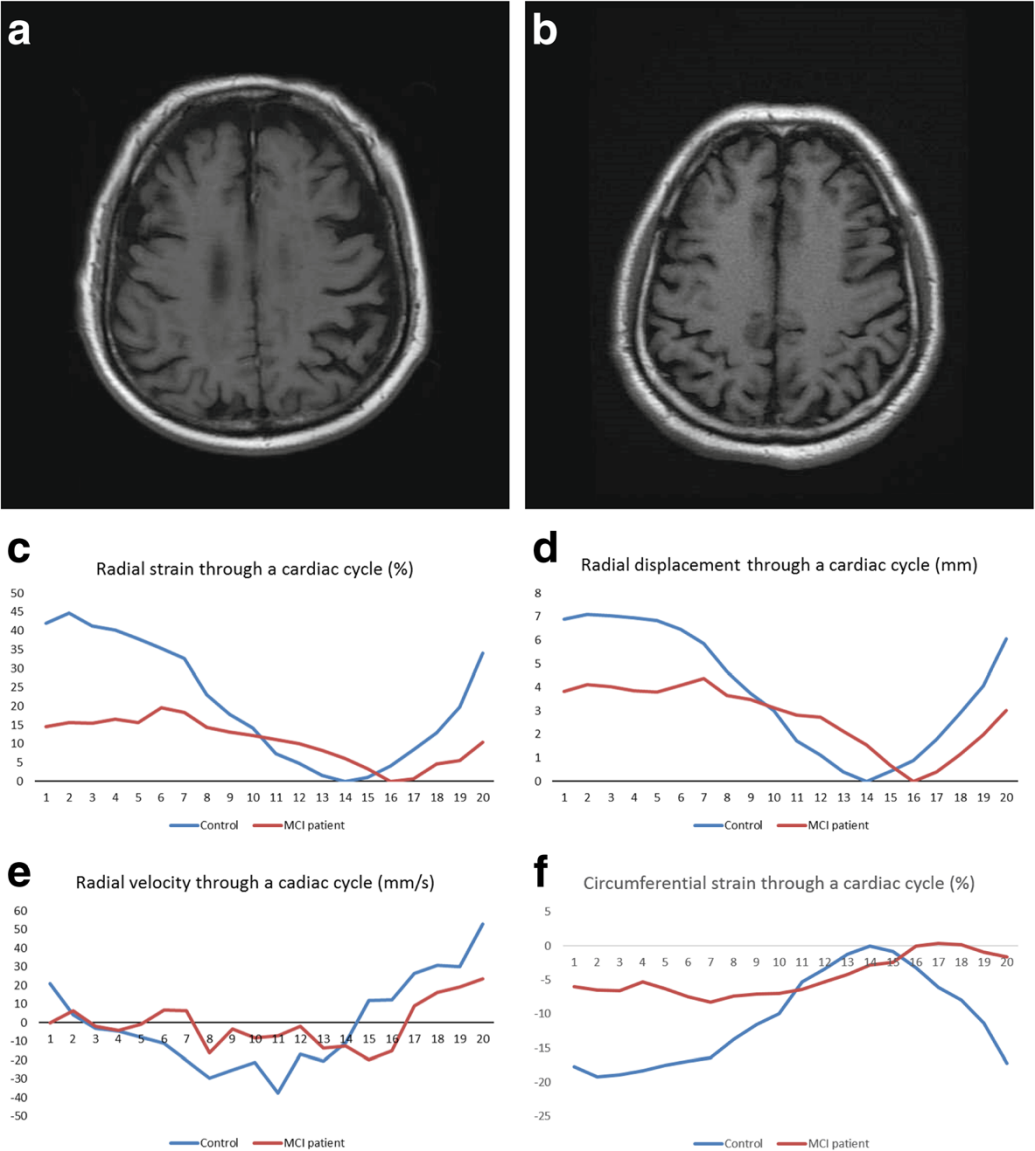
Comparisons of an MCI patient and a healthy control in manifestations of head MRI and regional myocardial motion patterns. **a** Participant X, a 69 years old female with MCI. Head MRI (T1WI) shows serious brain atrophy. GCA score is 2. **b** Participant Y, a 69 years old female control. Head MRI (T1WI) shows mild brain atrophy. GCA score is 1. **c** Time-resolved curves show that participant X has a lower radial strain than participant Y. **d** Time-resolved curves show that participant X has a lower peak displacement than participant Y. **e** Time-resolved curves show that participant X has a lower peak velocity (in systole and diastole) than participant Y. **f** Time-resolved curves show that participant X has a lower circumferential strain (in systole and diastole) than participant Y

There were no significant linear correlations between GCA scores and regional myocardial motion indices in 22 participants. Figure 3 (a -h) shows two typical cases (an MCI patient and a healthy control) with different GCA scores and regional myocardial motion patterns through a single cardiac cycle.

Good inter-observer agreement between the two reviewers for GCA grading in all 22 cases was found (ICC = 0.807). Bland-Altman plots showed small intra- and inter-observer variations in measuring peak strain at the radial, circumferential and long-axis directions; see Additional file 1.

## Discussion

In the present study, we quantitatively described MRI-derived regional myocardial motion patterns from multiple aspects in MCI patients. Compared to sex- and age-matched healthy controls, MCI patients had lower peak regional myocardial strain, strain rate, and velocity in certain directions or time phases. These findings suggest that regional myocardial motion indices, as early indicators of cardiovascular health, are associated with cognitive decline.

Although cognitive decline can be the result of various brain disorders, including tumors, strokes, trauma, infections, neurotoxicity and inherited genetic factors, AD accounts for 60 to 80% cognitive impairments in older adults [10]. Almost all AD cases would experience MCI. Some MCI patients would then progress to the advanced stage of AD: AD related dementia (ADRD). Unfortunately, there are no currently effective medications or devices that can be used to prevent or treat AD. On the other hand, CVD is also an age-related condition. The existence of CVDs have been closely linked to the development of MCI and dementia [11]. Heart failure, the end stage of heart damage, has been related to cognitive impairments in patients with CVDs [12]. In a cohort study conducted by Robert et al., a total of 2719 participants were followed for 15 months and evaluated for cognitive function using the Clinical Dementia Rating scale. The authors found that CVDs resulted in an increased risk of MCI (risk ratio, 1.77 [95% confidence interval, 1.16–2.72]) [13]. Rosano et al. demonstrated that the coronary calcium score, a traditional predictor of coronary heart disease (CHD), was related to the incidence of dementia [14]. In addition, Jefferson et al. found that cardiac index was related to information processing speed (*p* = 0.02), brain volume (*p* = 0.03) and lateral ventricular volume (*p* = 0.048) [15]. However, positive correlations between subclinical CVDs and AD are still not consistent. A double-blind, placebo-controlled trial, the Hypertension in the very Elderly Trial Cognitive Function Assessment (HYVET-COG) study, reported that anti-hypertension (HTN) treatment with indapamide (1.5 mg/d) in the elderly (> 80 years old) did not statistically reduce the incidence of dementia [16]. McGuinness et al. conducted a meta-analysis based on three randomized, double-blind, placebo-controlled trials for the assessment of anti-HTN therapies. However, the authors were unable to confirm that BP decreases in HTN patients could lower the incidence of cognitive impairment or AD [17]. *Different interpretations of these conflicting results generated discussion regarding whether “a healthy heart will result in a better brain”.* Hence*,* there is no conclusive evidence on whether preventing or treating CVDs benefits AD management.

*Recent studies have shown that CVDs might have a long-standing effect on the brain and therefore recommended early cardiovascular treatment/prevention to benefit the brain* [1]*. However,* current cardiovascular indices presenting global cardiac function, are insufficient for determining AD risk. Therefore, accurate description of regional myocardial motion patterns has high potential to provide critical information for assessing subclinical CVDs and their potential effects on the development of AD. Impaired regional myocardial motion patterns can be observed without a decrease in LVEF in asymptomatic subjects with prominent cardiovascular risk conditions [18–22]. Kresge et al. found that the global longitudinal strain (GLS), a cardiac motion index reflecting the shortening of the entire LV during a cardiac cycle is related to potential cognitive decline in older participants with normal LVEF [23]. *In the present study, we further demonstrated that regional myocardial motions indices from the LV were different between MCI patients and healthy controls while there was no significant difference in LVEF. More interestingly, differences in motion indices accompanied divergences in GCA scores in the two participant groups despite there being no direct linear correlations between them. T*he connections among *cardiac MRI, GCA scores and MCI suggest inherent correlations between the existence of subclinical CVDs and neurodegenerative changes. Although further investigations are needed to determine t*he mechanisms underlying our observations, *these cardiac imaging biomarkers* have the potential to inform the development of a cost-efficient prevention/treatment strategy simultaneously targeting CVD and AD, the two prominent threats to aging populations.

The present pilot study had limitations. First, we had a small sample size due to strict enrollment criteria for MCI patients. Therefore, we could only balance prominent cardiovascular risks, such as diabetes mellitus (DM), HTN, and body mass index (BMI), in data analysis. For the same reason, we were unable to distinguish subtypes of MCI (amnestic and nonamnestic) in the present study. Second, according to current cardiac MRI techniques, which might require frequent breath holding during image acquisition, it was difficult to perform cardiac MRI on patients with dementia. As a result, we had to intensively exclude dementia patients. Third, we were unable to acquire regional myocardial motion patterns from the right ventricle (RV) because the thin wall of RV substantially affects the contour drawing and therefore affect the accuracy of motion measurements. Fourth, the aim of the present study was to evaluate the clinical value of regional myocardial motion indices. Therefore, we only ran regular T1WI and T2WI on the participants to obtain GCA despite there being other MRI methods that can represent brain health from other aspects. Fifth, we were unable to determine a causal correlation between changes of regional myocardial motion and cognitive decline. A large-scale, longitudinal study has been designed to further assess the clinical value of cardiovascular imaging biomarkers in the management of AD.

## Conclusion

MRI-derived regional myocardial strain, strain rate and velocity were found to be different between MCI patients and healthy controls. Regional myocardial motion indices have the potential to become novel quantitative imaging biomarkers for representing the risk of neurodegenerative disorders, such as AD.

## Additional file

Additional file 1: Contain Bland-Altman plots for variations in measured regional myocardial motion indices. (DOCX 349 kb)

## Abbreviations

AD: Alzheimer’s disease
CVD: Cardiovascular disease
FOV: Field of view
FSE: Fast spin echo
GCA: Global cortical atrophy
LVEF: Left ventricular ejection fraction
MCI: Mild cognitive impairment
MRI: Magnetic resonance imaging
QC: Quality control
ROI: Region of interest
