# Supplementary material for: Regional myocardial motion in patients with mild cognitive impairment: a pilot study
Source: BMC Cardiovasc Disord. 2018 May 2;18:79. doi: 10.1186/s12872-018-0824-2 (PMC5932804; doi:10.1186/s12872-018-0824-2)
Supplement: Supplementary file 1 — Contain Bland-Altman plots for variations in measured regional myocardial motion indices. (DOCX 349 kb) [file 12872_2018_824_MOESM1_ESM.docx]

**Additional file 1**

**Figure 1 (a-x)**


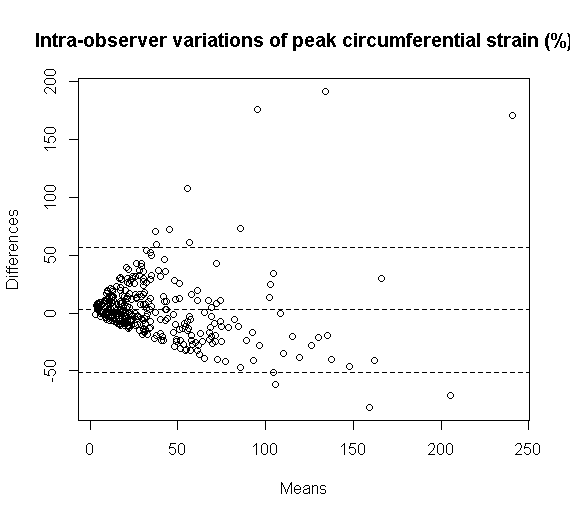


**Intra-observer variations of peak radial strain (%)**

**Figure 1a Intra-observer variations of peak radial strain**


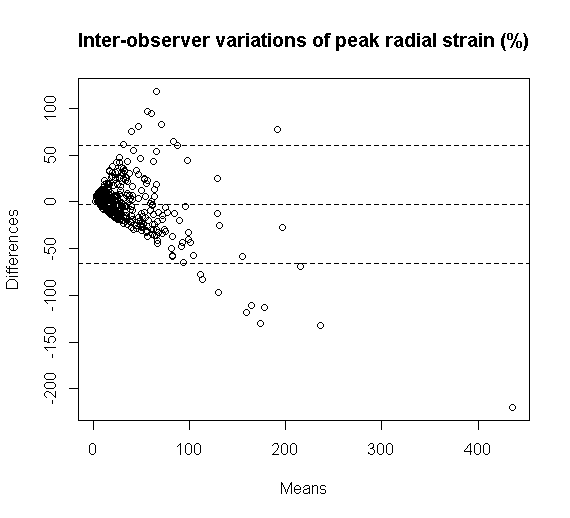


Figure 1b Inter-observer variations of peak radial strain


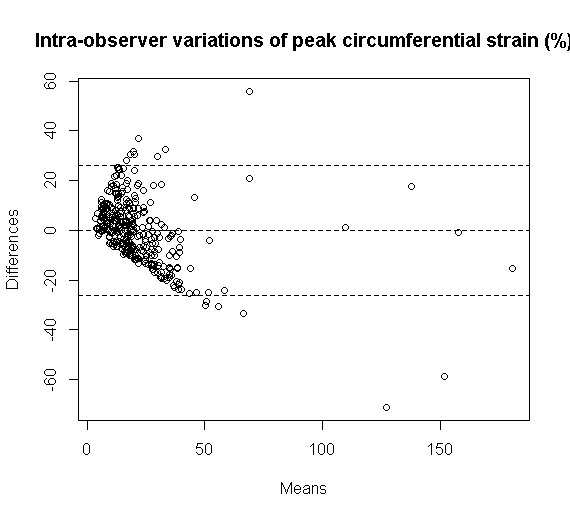


Figure 1c Intra-observer variations of peak circumferential strain


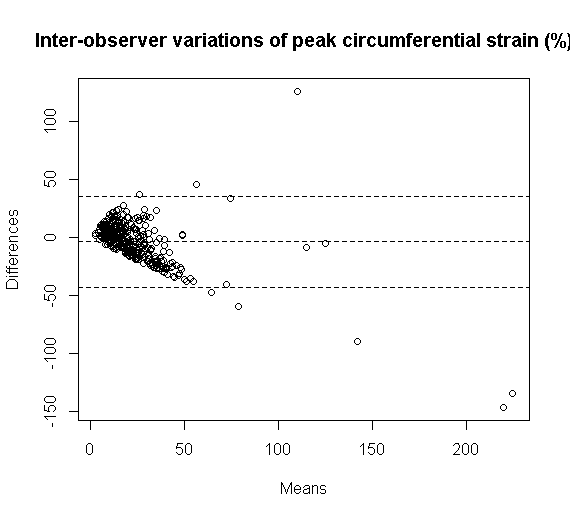


Figure 1d Inter-observer variations of peak circumferential strain


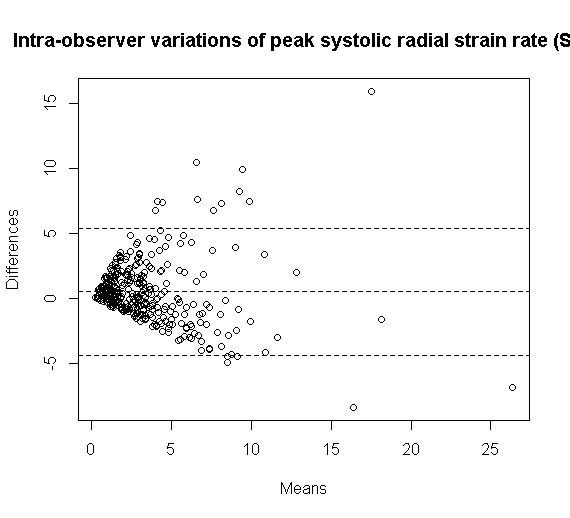


Figure 1e Intra-observer variations of peak radial strain rate


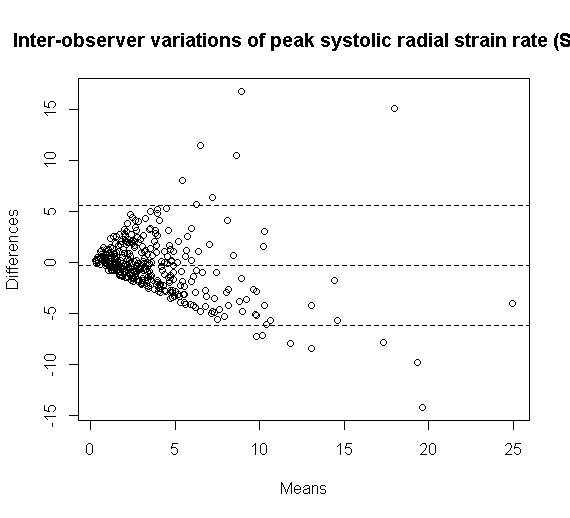


Figure 1f Inter-observer variations of peak systolic radial strain rate

**Intra-observer variations of peak systolic**

**circumferential strain rate (%)**


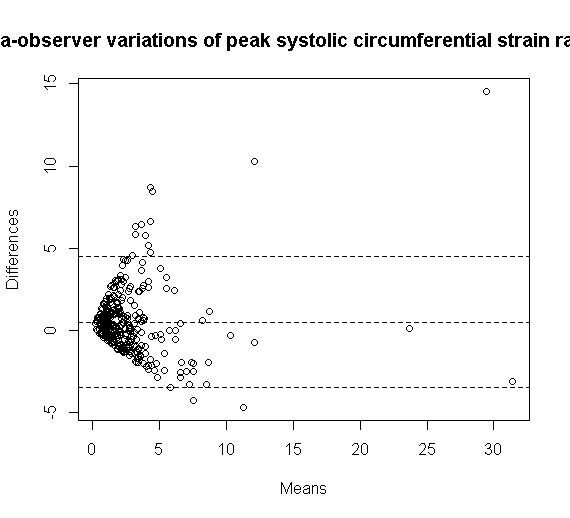


Figure 1g **Intra-observer variations of peak systolic circumferential strain rate**

**Inter-observer variations of peak systolic**

**circumferential strain rate (%)**


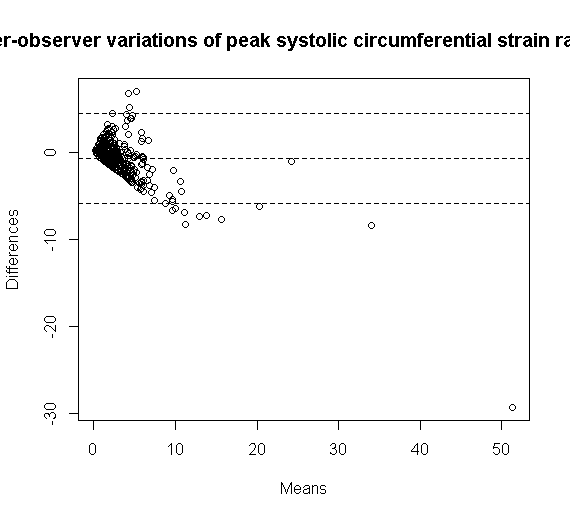


Figure 1h **Inter-observer variations of peak systolic circumferential strain rate**

**
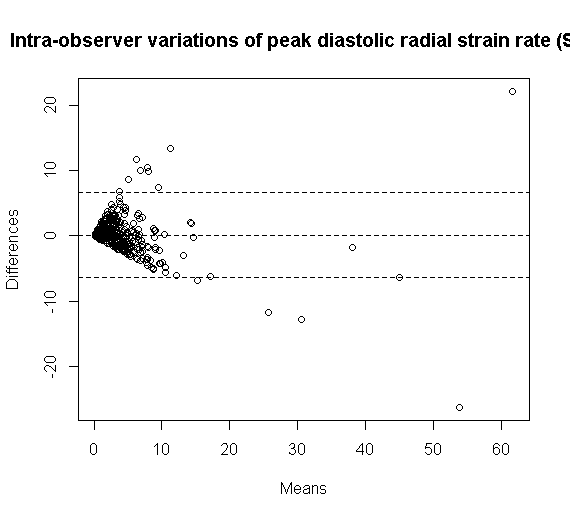
**

**Figure 1i Intra-observer variations of peak diastolic radial strain rate**

**
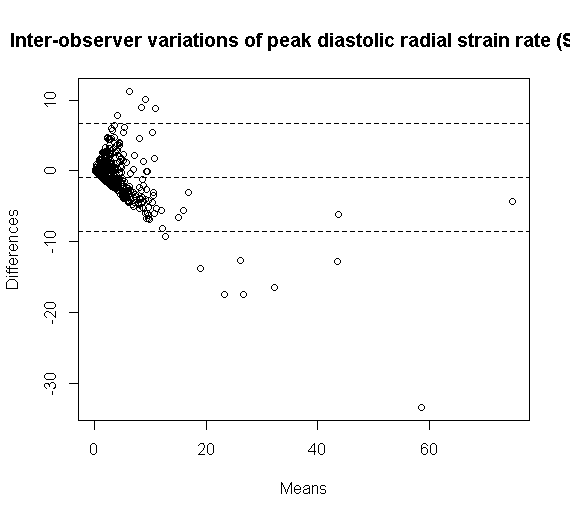
**

**Figure 1j Inter-observer variations of peak diastolic radial strain rate**

**Intra-observer variations of peak diastolic**

**circumferential strain rate (%)**

**
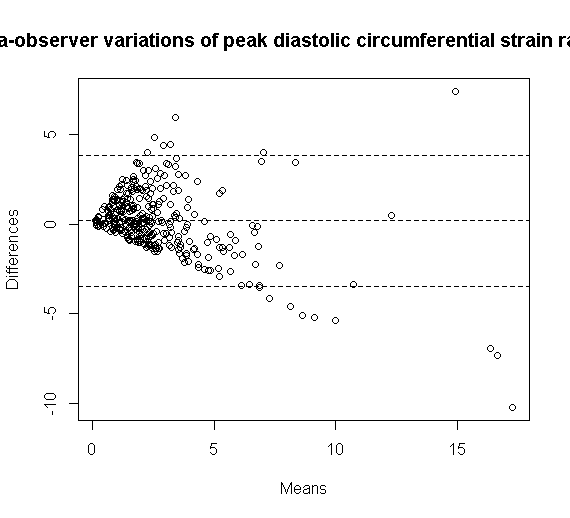
**

**Figure 1k Intra-observer variations of peak diastolic circumferential strain rate**

**Inter-observer variations of peak diastolic**

**circumferential strain rate (%)**

**
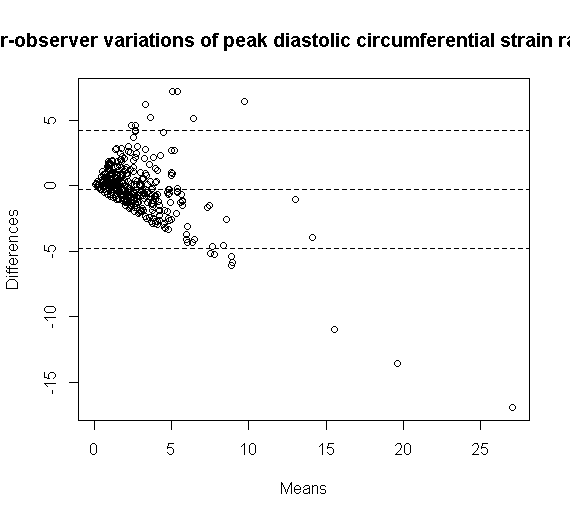
**

**Figure 1l Inter-observer variations of peak diastolic circumferential strain rate**

**
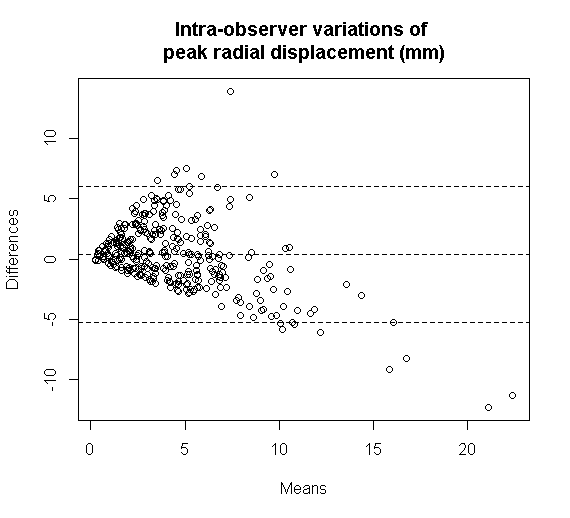
**

**Figure 1m Intra-observer variations of peak radial displacement**

**
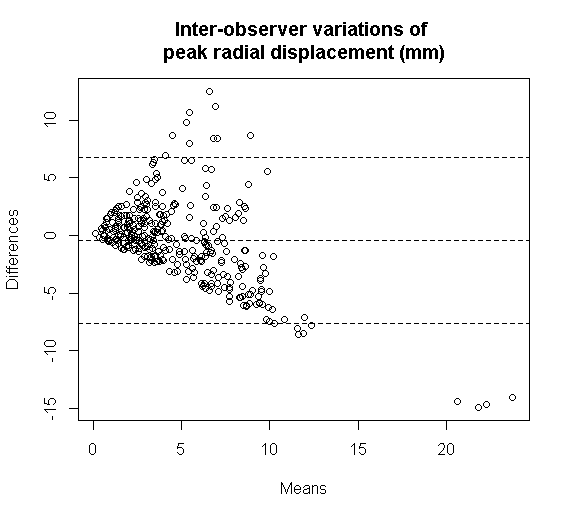
**

**Figure 1n Inter-observer variations of peak radial displacement**

**
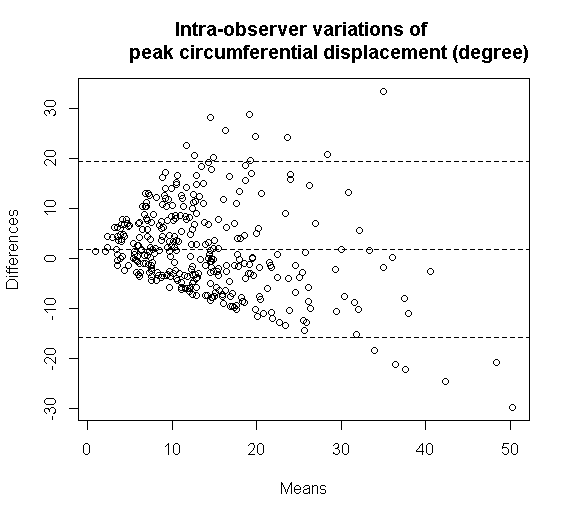
**

**Figure 1o Intra-observer variations of peak circumferential displacement**

**
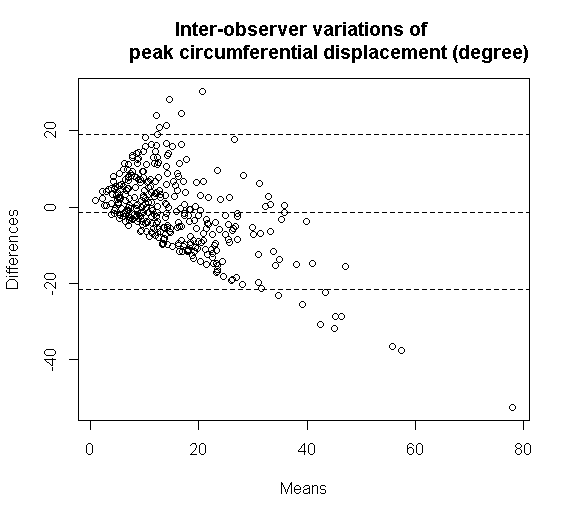
**

**Figure 1p Inter-observer variations of peak circumferential displacement**

**
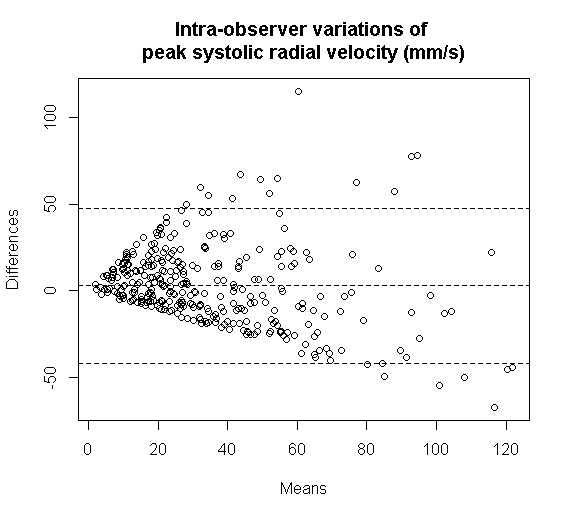
**

**Figure 1q Intra-observer variations of peak systolic radial velocity**

**
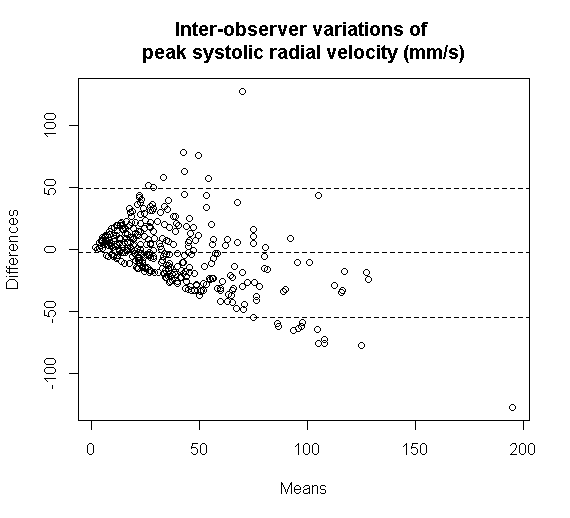
**

**Figure 1r Inter-observer variations of peak systolic radial velocity**

**
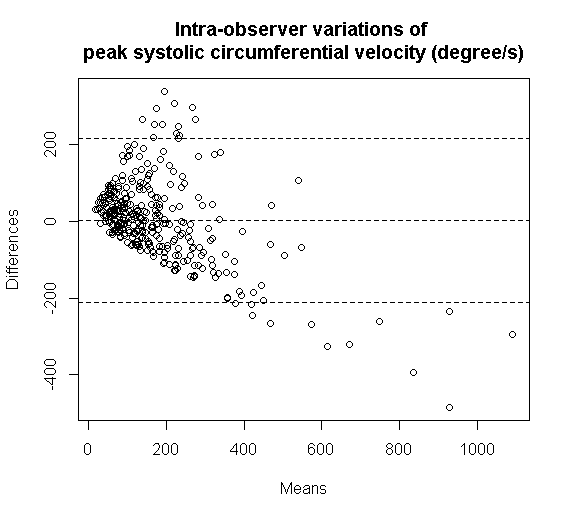
**

**Figure 1s Intra-observer variations of peak systolic circumferential velocity**

**
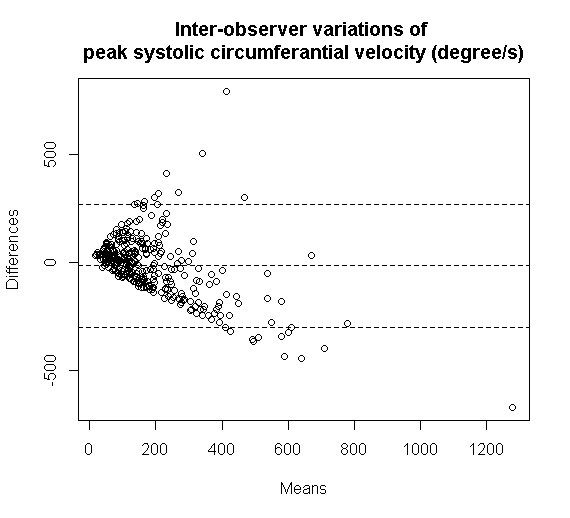
**

**Figure 1t Inter-observer variations of peak systolic circumferential velocity**

**
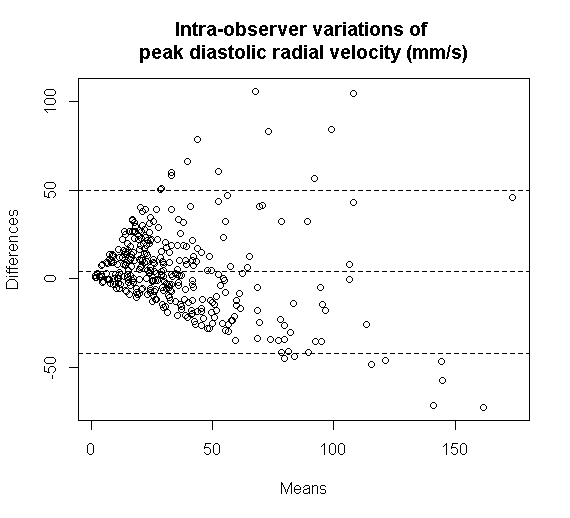
**

**Figure 1u Intra-observer variations of peak diastolic radial velocity**

**
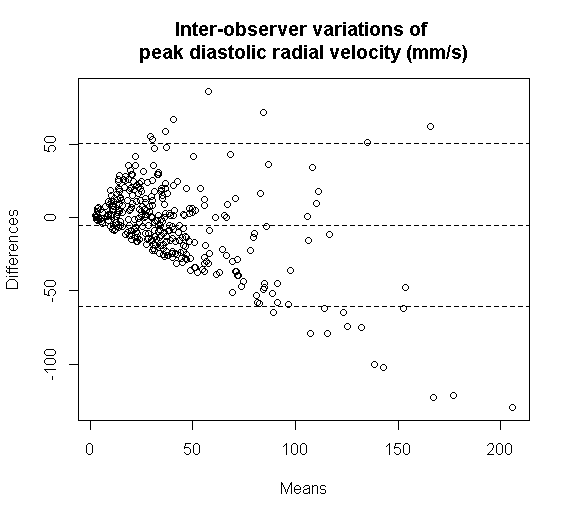
**

**Figure 1v Inter-observer variations of peak diastolic radial velocity**

**
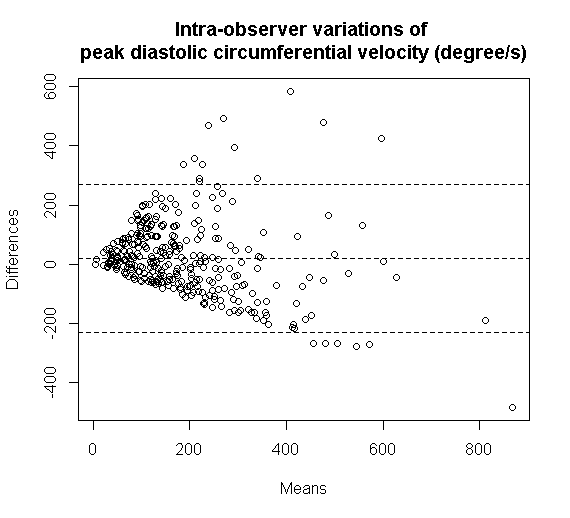
**

**Figure 1w Intra-observer variations of peak diastolic circumferential velocity**

**
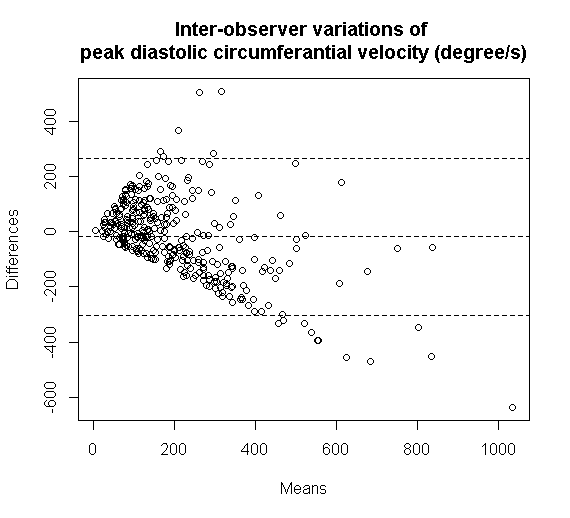
**

**Figure 1x Inter-observer variations of peak diastolic circumferential velocity**
